# 4-Anilino-1-benzyl­piperidine-4-carbo­nitrile

**DOI:** 10.1107/S1600536808009136

**Published:** 2008-04-16

**Authors:** Kiran K. Allam, Frank R. Fronczek, M. Graça H. Vicente

**Affiliations:** a Department of Chemistry, Louisiana State University, Baton Rouge, LA 70803-1804, USA

## Abstract

The title mol­ecule, C_19_H_21_N_3_, an important precursor in the synthesis of porphyrin–fentanyl conjugates, has its piperidine ring in the chair conformation, with endocyclic torsion-angle magnitudes in the range 53.26 (8)–60.63 (9)°. The C N group is axial, while the CH_2_Ph and NHPh groups are equatorial. The NH group does not engage in strong hydrogen bonding, but forms an inter­molecular N—H⋯N inter­action.

## Related literature

For background literature, see: Barth *et al.* (2005 ▶); Deguchi *et al.* (2004 ▶); Henriksen *et al.* (2005 ▶); Terasaki *et al.* (2003 ▶); Vicente, (2001 ▶). For a related structure, see: Brine *et al.* (1994 ▶).
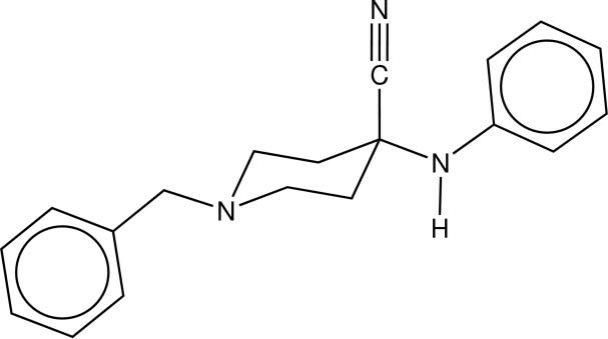


## Experimental

### 

#### Crystal data

C_19_H_21_N_3_
*M* *_r_* = 291.39
Monoclinic, 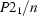

*a* = 9.7718 (13) Å
*b* = 10.0415 (14) Å
*c* = 15.9519 (15) Å
β = 94.532 (9)°
*V* = 1560.4 (3) Å^3^
*Z* = 4
Mo *K*α radiation
μ = 0.07 mm^−1^
*T* = 90 K
0.37 × 0.25 × 0.23 mm

#### Data collection

Nonius KappaCCD diffractometer with an Oxford Cryosystems Cryostream cooler
Absorption correction: none
24180 measured reflections
6842 independent reflections
5189 reflections with *I* > 2σ(*I*)
*R*_int_ = 0.025

#### Refinement

*R*[*F* ^2^ > 2σ(*F* ^2^)] = 0.044
*wR*(*F* ^2^) = 0.124
*S* = 1.03
6842 reflections
203 parameters
H atoms treated by a mixture of independent and constrained refinement
Δρ_max_ = 0.49 e Å^−3^
Δρ_min_ = −0.27 e Å^−3^

### 

Data collection: *COLLECT* (Nonius, 2000 ▶); cell refinement: *DENZO* and *SCALEPACK* (Otwinowski & Minor, 1997 ▶); data reduction: *DENZO* and *SCALEPACK*; program(s) used to solve structure: *SIR97* (Altomare *et al.*, 1999 ▶); program(s) used to refine structure: *SHELXL97* (Sheldrick, 2008 ▶); molecular graphics: *ORTEP-3 for Windows* (Farrugia, 1997 ▶); software used to prepare material for publication: *SHELXL97*.

## Supplementary Material

Crystal structure: contains datablocks global, I. DOI: 10.1107/S1600536808009136/om2222sup1.cif

Structure factors: contains datablocks I. DOI: 10.1107/S1600536808009136/om2222Isup2.hkl

Additional supplementary materials:  crystallographic information; 3D view; checkCIF report

## Figures and Tables

**Table 1:** Hydrogen-bond geometry (Å, °)

*D*—H⋯*A*	*D*—H	H⋯*A*	*D*⋯*A*	*D*—H⋯*A*
N2—H2*N*⋯N3^i^	0.847 (14)	2.756 (13)	3.5044 (12)	148.2 (11)

Symmetry code: (i) 
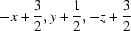
.

**Fig. 1.**
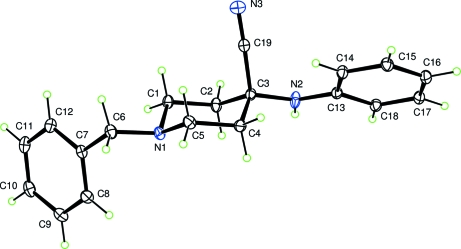
Ellipsoids at the 50% level, with H atoms having arbitrary radius.

## supplementary crystallographic information

### Comment

1-Benzyl-4-phenylamino-4-piperidinecarbonitrile is an important precursor in the synthesis of porphyrin-fentanyl conjugates that might be used as sensitizers in photodynamic therapy (PDT) (Vicente, 2001) and/or in boron neutron capture therapy (BNCT) (Barth *et al.*, 2005) of brain tumors. One of the obstacles for treating brain tumors using chemotherapy is the presence of the blood brain barrier (BBB), which protects the central nervous system from drugs and endogenous molecules (Terasaki *et al.*, 2003). Porphyrin-fentanyl conjugates could potentially cross the BBB due to the affinity of fentanyl derivatives for the opioid receptors highly expressed in the BBB (Deguchi *et al.*, 2004), and thus selectively accumulate within brain tumors.

The structure of the title compound is shown in Fig 1. The piperidine ring is in the chair conformation, with the C≡N group axial and the CH_2_Ph and NHPh groups equatorial. The conformation of the CH_2_Ph group with respect to the piperidine is described by torsion angles C5—N1—C6—C7 - 168.52 (6) and N1—C6—C7—C8 62.28 (10)°. The conformation of the NHPh group with respect to the piperidine is described by torsion angles C2—C3—N2—C13 179.19 (8) and C3—N2—C13—C18 172.56 (8)°. The pyramidal nature of N1 can be seen by the near-tetrahedral C—N1—C angles, which all fall within the narrow range 110.18 (6) - 110.76 (7)°, such that N1 lies 0.465 (1) Å from the plane defined by C1, C5, and C6.

Despite the presence of both a potential hydrogen-bond donor and potential hydrogen-bond acceptors, the compound exhibits no strong hydrogen bonding, nor any short C—H···N interactions. The nearest distance of the N—H group to a hydrogen-bond acceptor is to nitrile N3^i^ (at i = 3/2 - *x*, 1/2 + *y*, 3/2 - *z*), having N2···N3^i^ distance 3.5044 (12) Å, H2N··· N3^i^ distance 2.756 (13) Å, and angle about H2N 148.2 (11)°.

### Experimental

The title compound was prepared in 89.5% yield from *N*-benzyl-4-piperidone, using an optimized procedure from that previously published (Henriksen *et al.*, 2005), as follows. To a 100 ml round-bottom flask under an argon atmosphere were added *N*-benzyl-4-piperidone (1.89 g, 10 mmol), aniline (3.7 g, 40 mmol), KCN (2.6 g, 40 mmol) and dry dichloromethane (40 ml). The reaction mixture was cooled to 0° C and stirred under argon for 20 minutes. Acetic acid (1.8 g, 30 mmol) was added to the reaction mixture over a period of 10 minutes and the final mixture heated at 50° C for 24 h. After cooling to room temperature the reaction mixture was poured into crushed ice (50 g), neutralized with 25% aqueous NaOH and the pH of the mixture was adjusted to about 10 using 40% aqueous K_2_CO_3_. The organic phase was collected and the water layer was extracted with dichloromethane (2 *x* 25 ml). The organic extracts were dried over anhydrous sodium bicarbonate and concentrated under reduced pressure to give a yellow solid. The yellow crystals were purified by re-crystallization from dichloromethane/hexane to give 2.6 g (89.5%) yield of colorless crystals. Spectroscopic analysis, ^1^H NMR (250 MHz, CDCl_3_): 1.92 (td, J_1_= 3.6 Hz, J_2_ = 10.8 Hz, 2H, CH_2_), 2.29–2.53 (m, 4H, CH_2_), 2.79–2.85 (m, 2H, CH_2_), 3.56 (s, 2H, CH_2_Ph), 3.64 (s, 1H, NH), 6.88–6.95 (m, 3H, ArH), 7.20–7.33 (m, 7H, ArH). ^13^C NMR (CDCl_3_): 36.09, 49.27, 59.06, 62.58, 117.78, 120.93, 127.26, 128.96, 129.00, 129.31, 134.02, 138.00, 143.29. MS MALDI-TOF *m/z* 290.9 (*M*^+^).

### Refinement

H atoms on C were placed in idealized positions with C—H distances 0.95 - 0.99 Å and thereafter treated as riding. Coordinates of the N—H hydrogen atom were refined. *U*_iso_ for H was assigned as 1.2 times *U*_eq_ of the attached atoms.

### Crystal data

**Table:**

C_19_H_21_N_3_	*F*_000_ = 624
*M**_r_* = 291.39	*D*_x_ = 1.240 Mg m^−^^3^
Monoclinic, *P*2_1_/*n*	Mo *K*α radiation λ = 0.71073 Å
Hall symbol: -P 2yn	Cell parameters from 5228 reflections
*a* = 9.7718 (13) Å	θ = 2.5–35.0º
*b* = 10.0415 (14) Å	µ = 0.07 mm^−^^1^
*c* = 15.9519 (15) Å	*T* = 90 K
β = 94.532 (9)º	Fragment, colorless
*V* = 1560.4 (3) Å^3^	0.37 × 0.25 × 0.23 mm
*Z* = 4	

### Data collection

**Table:**

Nonius KappaCCD diffractometer with an Oxford Cryosystems Cryostream cooler	5189 reflections with *I* > 2σ(*I*)
Radiation source: fine-focus sealed tube	*R*_int_ = 0.025
Monochromator: graphite	θ_max_ = 35.0º
*T* = 90 K	θ_min_ = 2.5º
ω scans with κ offsets	*h* = −15→15
Absorption correction: none	*k* = −12→16
24180 measured reflections	*l* = −25→25
6842 independent reflections	

### Refinement

**Table:**

Refinement on *F*^2^	Hydrogen site location: inferred from neighbouring sites
Least-squares matrix: full	H atoms treated by a mixture of independent and constrained refinement
*R*[*F*^2^ > 2σ(*F*^2^)] = 0.044	*w* = 1/[σ^2^(*F*_o_^2^) + (0.0605*P*)^2^ + 0.3181*P*] where *P* = (*F*_o_^2^ + 2*F*_c_^2^)/3
*wR*(*F*^2^) = 0.124	(Δ/σ)_max_ = 0.001
*S* = 1.04	Δρ_max_ = 0.49 e Å^−^^3^
6842 reflections	Δρ_min_ = −0.27 e Å^−^^3^
203 parameters	Extinction correction: SHELXL97 (Sheldrick, 2008), Fc^*^=kFc[1+0.001xFc^2^λ^3^/sin(2θ)]^-1/4^
Primary atom site location: structure-invariant direct methods	Extinction coefficient: 0.0081 (18)
Secondary atom site location: difference Fourier map	

### Special details

**Table:**

Geometry. All e.s.d.'s (except the e.s.d. in the dihedral angle between two l.s. planes) are estimated using the full covariance matrix. The cell e.s.d.'s are taken into account individually in the estimation of e.s.d.'s in distances, angles and torsion angles; correlations between e.s.d.'s in cell parameters are only used when they are defined by crystal symmetry. An approximate (isotropic) treatment of cell e.s.d.'s is used for estimating e.s.d.'s involving l.s. planes.
Refinement. Refinement of *F*^2^ against ALL reflections. The weighted *R*-factor *wR* and goodness of fit *S* are based on *F*^2^, conventional *R*-factors *R* are based on *F*, with *F* set to zero for negative *F*^2^. The threshold expression of *F*^2^ > σ(*F*^2^) is used only for calculating *R*-factors(gt) *etc*. and is not relevant to the choice of reflections for refinement. *R*-factors based on *F*^2^ are statistically about twice as large as those based on *F*, and *R*- factors based on ALL data will be even larger.

### Fractional atomic coordinates and isotropic or equivalent isotropic displacement parameters (Å^2^)

**Table:**

	*x*	*y*	*z*	*U*_iso_*/*U*_eq_	
N1	0.43216 (7)	0.37118 (8)	0.73394 (4)	0.01247 (13)	
N2	0.68233 (8)	0.66885 (8)	0.63306 (4)	0.01659 (14)	
H2N	0.6447 (13)	0.7350 (14)	0.6548 (8)	0.020*	
N3	0.86941 (8)	0.40176 (9)	0.70892 (5)	0.01981 (15)	
C1	0.53227 (8)	0.44146 (9)	0.79097 (4)	0.01280 (14)	
H1A	0.6124	0.3830	0.8050	0.015*	
H1B	0.4903	0.4628	0.8438	0.015*	
C2	0.57970 (8)	0.56932 (9)	0.75123 (5)	0.01372 (14)	
H2A	0.6475	0.6144	0.7910	0.016*	
H2B	0.5002	0.6298	0.7403	0.016*	
C3	0.64507 (8)	0.54198 (8)	0.66810 (5)	0.01146 (13)	
C4	0.54095 (8)	0.45965 (9)	0.61165 (5)	0.01336 (14)	
H4A	0.4602	0.5158	0.5947	0.016*	
H4B	0.5837	0.4328	0.5600	0.016*	
C5	0.49370 (8)	0.33556 (9)	0.65622 (5)	0.01364 (15)	
H5A	0.4256	0.2869	0.6185	0.016*	
H5B	0.5731	0.2759	0.6695	0.016*	
C6	0.38149 (8)	0.25204 (9)	0.77503 (5)	0.01443 (14)	
H6A	0.4604	0.1982	0.7983	0.017*	
H6B	0.3273	0.1971	0.7329	0.017*	
C7	0.29300 (8)	0.28909 (9)	0.84504 (5)	0.01256 (14)	
C8	0.17137 (8)	0.35979 (9)	0.82626 (5)	0.01463 (15)	
H8	0.1458	0.3852	0.7698	0.018*	
C9	0.08730 (9)	0.39339 (9)	0.88947 (5)	0.01645 (16)	
H9	0.0047	0.4414	0.8760	0.020*	
C10	0.12395 (9)	0.35679 (10)	0.97276 (5)	0.01752 (16)	
H10	0.0655	0.3777	1.0157	0.021*	
C11	0.24640 (10)	0.28969 (9)	0.99214 (5)	0.01758 (16)	
H11	0.2730	0.2665	1.0488	0.021*	
C12	0.33101 (9)	0.25597 (9)	0.92851 (5)	0.01539 (15)	
H12	0.4149	0.2102	0.9423	0.018*	
C13	0.74564 (8)	0.68867 (8)	0.55887 (5)	0.01178 (14)	
C14	0.80105 (8)	0.58537 (8)	0.51281 (5)	0.01315 (14)	
H14	0.7935	0.4957	0.5308	0.016*	
C15	0.86740 (8)	0.61438 (9)	0.44045 (5)	0.01405 (14)	
H15	0.9037	0.5437	0.4094	0.017*	
C16	0.88109 (8)	0.74472 (9)	0.41323 (5)	0.01466 (15)	
H16	0.9273	0.7636	0.3644	0.018*	
C17	0.82588 (8)	0.84752 (9)	0.45882 (5)	0.01452 (15)	
H17	0.8344	0.9371	0.4408	0.017*	
C18	0.75859 (8)	0.82007 (9)	0.53034 (5)	0.01298 (14)	
H18	0.7208	0.8911	0.5604	0.016*	
C19	0.77255 (8)	0.46283 (9)	0.68964 (5)	0.01285 (14)	

### Atomic displacement parameters (Å^2^)

**Table:**

	*U*^11^	*U*^22^	*U*^33^	*U*^12^	*U*^13^	*U*^23^
N1	0.0132 (3)	0.0145 (3)	0.0100 (2)	−0.0015 (2)	0.0032 (2)	−0.0008 (2)
N2	0.0258 (4)	0.0095 (3)	0.0161 (3)	0.0039 (3)	0.0115 (3)	0.0025 (2)
N3	0.0166 (3)	0.0209 (4)	0.0217 (3)	0.0027 (3)	0.0001 (2)	0.0017 (3)
C1	0.0158 (3)	0.0133 (3)	0.0096 (3)	−0.0012 (3)	0.0027 (2)	0.0001 (3)
C2	0.0178 (3)	0.0127 (3)	0.0113 (3)	0.0002 (3)	0.0056 (2)	−0.0002 (3)
C3	0.0131 (3)	0.0106 (3)	0.0111 (3)	0.0017 (3)	0.0034 (2)	0.0015 (2)
C4	0.0125 (3)	0.0182 (4)	0.0095 (3)	0.0011 (3)	0.0016 (2)	0.0012 (3)
C5	0.0137 (3)	0.0168 (4)	0.0106 (3)	−0.0015 (3)	0.0024 (2)	−0.0024 (3)
C6	0.0155 (3)	0.0131 (3)	0.0152 (3)	−0.0009 (3)	0.0050 (2)	−0.0007 (3)
C7	0.0133 (3)	0.0121 (3)	0.0126 (3)	−0.0022 (3)	0.0032 (2)	−0.0003 (3)
C8	0.0135 (3)	0.0170 (4)	0.0134 (3)	−0.0017 (3)	0.0016 (2)	−0.0013 (3)
C9	0.0132 (3)	0.0178 (4)	0.0187 (3)	−0.0017 (3)	0.0036 (3)	−0.0033 (3)
C10	0.0200 (4)	0.0168 (4)	0.0167 (3)	−0.0048 (3)	0.0080 (3)	−0.0025 (3)
C11	0.0250 (4)	0.0146 (4)	0.0136 (3)	−0.0030 (3)	0.0046 (3)	0.0016 (3)
C12	0.0183 (3)	0.0130 (4)	0.0151 (3)	−0.0003 (3)	0.0024 (3)	0.0021 (3)
C13	0.0125 (3)	0.0117 (3)	0.0114 (3)	0.0011 (3)	0.0027 (2)	0.0014 (2)
C14	0.0157 (3)	0.0109 (3)	0.0134 (3)	0.0010 (3)	0.0042 (2)	0.0012 (3)
C15	0.0148 (3)	0.0150 (4)	0.0128 (3)	0.0007 (3)	0.0038 (2)	−0.0007 (3)
C16	0.0150 (3)	0.0163 (4)	0.0131 (3)	0.0002 (3)	0.0038 (2)	0.0022 (3)
C17	0.0152 (3)	0.0132 (4)	0.0154 (3)	0.0002 (3)	0.0032 (2)	0.0039 (3)
C18	0.0141 (3)	0.0115 (3)	0.0135 (3)	0.0012 (3)	0.0027 (2)	0.0009 (3)
C19	0.0139 (3)	0.0126 (3)	0.0123 (3)	−0.0011 (3)	0.0023 (2)	0.0003 (3)

### Geometric parameters (Å, °)

**Table:**

N1—C1	1.4636 (10)	C7—C12	1.3940 (11)
N1—C5	1.4644 (10)	C7—C8	1.3964 (12)
N1—C6	1.4688 (11)	C8—C9	1.3915 (11)
N2—C13	1.3924 (10)	C8—H8	0.9500
N2—C3	1.4491 (11)	C9—C10	1.3977 (12)
N2—H2N	0.847 (14)	C9—H9	0.9500
N3—C19	1.1492 (11)	C10—C11	1.3867 (13)
C1—C2	1.5203 (12)	C10—H10	0.9500
C1—H1A	0.9900	C11—C12	1.4004 (12)
C1—H1B	0.9900	C11—H11	0.9500
C2—C3	1.5414 (11)	C12—H12	0.9500
C2—H2A	0.9900	C13—C14	1.4042 (11)
C2—H2B	0.9900	C13—C18	1.4047 (12)
C3—C19	1.4946 (11)	C14—C15	1.3986 (11)
C3—C4	1.5443 (11)	C14—H14	0.9500
C4—C5	1.5242 (12)	C15—C16	1.3887 (12)
C4—H4A	0.9900	C15—H15	0.9500
C4—H4B	0.9900	C16—C17	1.3955 (12)
C5—H5A	0.9900	C16—H16	0.9500
C5—H5B	0.9900	C17—C18	1.3888 (11)
C6—C7	1.5119 (11)	C17—H17	0.9500
C6—H6A	0.9900	C18—H18	0.9500
C6—H6B	0.9900		
			
C1—N1—C5	110.18 (6)	C7—C6—H6B	109.4
C1—N1—C6	110.34 (6)	H6A—C6—H6B	108.0
C5—N1—C6	110.76 (7)	C12—C7—C8	118.96 (7)
C13—N2—C3	126.55 (7)	C12—C7—C6	121.47 (7)
C13—N2—H2N	118.2 (9)	C8—C7—C6	119.57 (7)
C3—N2—H2N	113.6 (9)	C9—C8—C7	120.60 (7)
N1—C1—C2	111.03 (6)	C9—C8—H8	119.7
N1—C1—H1A	109.4	C7—C8—H8	119.7
C2—C1—H1A	109.4	C8—C9—C10	120.23 (8)
N1—C1—H1B	109.4	C8—C9—H9	119.9
C2—C1—H1B	109.4	C10—C9—H9	119.9
H1A—C1—H1B	108.0	C11—C10—C9	119.43 (8)
C1—C2—C3	111.66 (7)	C11—C10—H10	120.3
C1—C2—H2A	109.3	C9—C10—H10	120.3
C3—C2—H2A	109.3	C10—C11—C12	120.29 (8)
C1—C2—H2B	109.3	C10—C11—H11	119.9
C3—C2—H2B	109.3	C12—C11—H11	119.9
H2A—C2—H2B	108.0	C7—C12—C11	120.44 (8)
N2—C3—C19	109.02 (7)	C7—C12—H12	119.8
N2—C3—C2	108.01 (7)	C11—C12—H12	119.8
C19—C3—C2	106.94 (6)	N2—C13—C14	123.69 (7)
N2—C3—C4	114.82 (6)	N2—C13—C18	117.84 (7)
C19—C3—C4	110.37 (7)	C14—C13—C18	118.43 (7)
C2—C3—C4	107.35 (6)	C15—C14—C13	120.06 (8)
C5—C4—C3	112.06 (6)	C15—C14—H14	120.0
C5—C4—H4A	109.2	C13—C14—H14	120.0
C3—C4—H4A	109.2	C16—C15—C14	121.12 (8)
C5—C4—H4B	109.2	C16—C15—H15	119.4
C3—C4—H4B	109.2	C14—C15—H15	119.4
H4A—C4—H4B	107.9	C15—C16—C17	118.91 (7)
N1—C5—C4	110.80 (7)	C15—C16—H16	120.5
N1—C5—H5A	109.5	C17—C16—H16	120.5
C4—C5—H5A	109.5	C18—C17—C16	120.59 (8)
N1—C5—H5B	109.5	C18—C17—H17	119.7
C4—C5—H5B	109.5	C16—C17—H17	119.7
H5A—C5—H5B	108.1	C17—C18—C13	120.88 (8)
N1—C6—C7	111.21 (7)	C17—C18—H18	119.6
N1—C6—H6A	109.4	C13—C18—H18	119.6
C7—C6—H6A	109.4	N3—C19—C3	177.73 (8)
N1—C6—H6B	109.4		
			
C5—N1—C1—C2	60.63 (9)	C12—C7—C8—C9	−1.83 (13)
C6—N1—C1—C2	−176.75 (6)	C6—C7—C8—C9	179.10 (8)
N1—C1—C2—C3	−58.68 (9)	C7—C8—C9—C10	0.10 (13)
C13—N2—C3—C19	63.33 (10)	C8—C9—C10—C11	1.61 (14)
C13—N2—C3—C2	179.19 (8)	C9—C10—C11—C12	−1.57 (14)
C13—N2—C3—C4	−61.08 (11)	C8—C7—C12—C11	1.86 (13)
C1—C2—C3—N2	177.84 (6)	C6—C7—C12—C11	−179.09 (8)
C1—C2—C3—C19	−64.94 (8)	C10—C11—C12—C7	−0.17 (14)
C1—C2—C3—C4	53.51 (8)	C3—N2—C13—C14	−9.65 (13)
N2—C3—C4—C5	−173.35 (7)	C3—N2—C13—C18	172.56 (8)
C19—C3—C4—C5	62.96 (8)	N2—C13—C14—C15	−177.68 (8)
C2—C3—C4—C5	−53.26 (8)	C18—C13—C14—C15	0.09 (12)
C1—N1—C5—C4	−59.98 (8)	C13—C14—C15—C16	0.65 (12)
C6—N1—C5—C4	177.65 (6)	C14—C15—C16—C17	−0.77 (12)
C3—C4—C5—N1	57.77 (8)	C15—C16—C17—C18	0.15 (12)
C1—N1—C6—C7	69.20 (8)	C16—C17—C18—C13	0.59 (12)
C5—N1—C6—C7	−168.52 (6)	N2—C13—C18—C17	177.20 (7)
N1—C6—C7—C12	−116.76 (9)	C14—C13—C18—C17	−0.70 (12)
N1—C6—C7—C8	62.28 (10)		

### Hydrogen-bond geometry (Å, °)

**Table:**

*D*—H···*A*	*D*—H	H···*A*	*D*···*A*	*D*—H···*A*
N2—H2N···N3^i^	0.847 (14)	2.756 (13)	3.5044 (12)	148.2 (11)

Symmetry codes: (i) −*x*+3/2, *y*+1/2, −*z*+3/2.
